# The Fourth International Dental Congress

**Published:** 1904-10

**Authors:** 


### Editorial.

---

### THE FOURTH INTERNATIONAL DENTAL CONGRESS.

THE Fourth International Dental Congress has passed into history, and those who took an active part in it have equally moved onward to their several destinations. It now remains for the dental periodicals of the world to give their readers, not able to be present, the general impressions of this great gathering of dentists.

This self-imposed task seems to be a difficult one. It was very evident to the writer during the sessions of this Congress, that no single mind could do exact justice to this meeting. Each one present studied it from his own point of observation, and this, doubtless, led in many instances to false impressions of the Congress as a whole.

The meeting convened at the "Coliseum" on Monday, the 29th of August, 1904. The auditorium of this building would comfortably seat two thousand five hundred persons, and when the time arrived for calling the meeting to order it was fully two-thirds full.

It had been evident for several days prior to the organization of the Congress that a political storm was in progress, and this assumed very unpleasant proportions as the time of the meeting approached. The cause of this was the old and ever discreditable American fight for offices. We use the word American with a full understanding of its meaning. It is believed that in no other country could this happen to the same extent; and while human nature is very much the same everywhere, in no other land is the desire for office so intense as in this country. The reason for this is quite apparent to the student of racial proclivities, but space and time will not permit a thorough consideration of this part of the subject. It is sufficient to say that this contention crystallized itself on two positions, —those of President and Secretary-General. The origin of this controversy dates back to the appointment of the Committee of Fifteen by the National Dental Association and the appointment of the Committee of Nine by the "Fédération Dentaire Internationale" at Stockholm. The story is too long and too unprofitable to be repeated here. The result was that the first session of the Congress witnessed the culmination of this strife for office that must have astonished the foreign delegates.

The meeting was opened by Howard J. Rogers, Director of Congresses, in an able address of welcome. He, at the close of this, acted as temporary president to effect a permanent organization. The nominations for President were Dr. H. J. Burkhart, of Batavia, N. Y., and Dr. C. N. Johnson, of Chicago, the former having been president of the Committee of Organization. For Secretary-General the nominations were Dr. E. C. Kirk, of Philadelphia, secretary of the Committee, and Dr. A. W. Harlan, of New York City. For days previous to this meeting, this contest had been fought over behind closed doors, with the result that at the opening there was a general suppressed excitement and much uncertainty felt as to the final result. The vote for both offices was taken by each member standing until the vote was counted. The result for President was in favor of Dr. Burkhart by a large majority. The position of Secretary-General was decided by a practically unanimous vote for Dr. E. C. Kirk. The introduction of foreign delegates then followed, with short speeches from each. This occupied the first session, and the sections began their work in the afternoon.

In the opinion of the writer, the Congress was not opened with the dignity belonging to a great international organization. Had the permanent officers been selected months ago it would have permitted the successful aspirant for the office of President to have prepared a fitting address. This, of course, could not be done under the method adopted, resulting in a very informal opening, and to some very unsatisfactory.

No attempt will be made in this article to report the proceedings. As before stated, the story can only be told through the reports from Sections and the published volumes.

If the success of a congress is based on numbers—eighteen hundred being registered—and papers presented, this must be regarded as fulfilling all requirements. The world was well represented,—Europe, China, Japan, Philippines, Australia, New Zealand, America, and the islands of the oceans,—England alone not being represented by native Britons. Subjects of Great Britain were active in the Congress, but the mother country was not represented by any of her prominent workers. We presume this is capable of explanation, but it was an unpleasant feature in the complexion of the Congress.

The value of the papers can be judged only by careful reading after publication, for it is surmised that not one in attendance was entirely satisfied with the reading under conditions present. The writer had access to a limited number, and, if an opinion can be based on these, it may be safe to say that the final report will be of very great value.

The clinics were very extensive and the chairman and his assistants deserve great credit for the work accomplished. They exceeded anything of the kind ever attempted in this country. Their value the writer must leave to others to determine. The time was so much occupied in other directions, that but little could be spared for these or the exhibits. Both of these evidently were of more intrinsic value to the members than the sections, for the former were at all times crowded while the latter were almost entirely neglected.

Was the Congress a success? This question will, it is presumed, be asked over and over again, and the answer each time will be one of uncertainty. With a very few it will be regarded as having met all expectations, but with the large majority it was a serious disappointment, if not practically a failure. To the latter class the writer belongs, and it is due that large body that the reasons for this opinion should be given. That the Congress failed to meet the highest anticipations of its members is not, by any means, remarkable, for this, probably, may be said of all aggregations of men; neither can the Committee of Organization be censured for any shortcomings, for it simply followed precedent in its energetic and laborious work, but that this meeting was very unsatisfactory was the almost universal verdict.

The reason for this was primarily due to the fact that the Congress was divided into sections. This may be necessary in great gatherings, similar to the National Medical Association of this country, from the impossibility of accommodating the diversity of interests in any one building, and to some extent this is true of congresses of international character in dentistry, but experience has demonstrated that at no general meeting was it shown that all the papers possible to read could not have been read and discussed there. This was quite fully demonstrated when almost the entire membership occupied the auditorium to listen to Professor W. D. Miller's paper. The section work was well tested at the Columbian Dental Congress, and proved very unsatisfactory there, and why it should have been adopted again at this Congress remains for future explanation. The result was disastrous. In but few sections, from personal observation, was there more than ten or fifteen present, and oftentimes not that number, and in some the attendance was limited to the officers of the section. One chairman of an important section informed the writer that he was obliged to have most of his papers read by title, as he had no audience to listen to them. This is a humiliating confession, but it is better to give the exact truth now, that future congresses may profit by this unfortunate experience.

The reason for this indifference is not far to seek. The great Exposition attracted many; others found more instruction, as they did interest, in the exhibits, and still others found the social intercourse in the halls a very great pleasure; but above and beyond all these reasons, there was the main fact that some one invited this International Dental Congress to meet in a building that was in itself sufficient to stamp failure upon this and every other congress of the series, providing, as in this, they must branch out from the main auditorium to the widely scattered rooms below and above. This building, as it now stands, is a blot upon the character of American civilization, and the expressions of indignation in regard to this were loud and prolonged, but at the same time equally useless. The local committee had tried to clean it, but all efforts in this direction were futile. This building is recognized in St. Louis as having passed its day of usefulness, and it is proposed to have it demolished after the Exposition has been closed. It remains an unsolved problem why the Exposition managers should have selected this building for the various congresses. Its deplorable condition must have been well known, and the certainty that this would reflect upon American character and American civilization should have been seriously considered. Those who attended some of the congresses of the Columbian Exposition of 1893 could not fail to draw comparisons very unfavorable to the situation in St. Louis in 1904. The acoustic properties of the building throughout were so bad that even in the smaller rooms with lofty ceilings the members were in a constant strain to hear the papers and discussions. The result was that this, combined with the aforesaid uncleanly condition, broke up the sections. Had all the meetings been held in the main auditorium, it would have been better, but even here the conditions described were so unsatisfactory that many left the meetings, finding it impossible to hear, and others remained under protest rather than give the appearance of neglecting the work of the Congress.

The social features consisted of a reception at the Missouri Building, Exposition grounds. This the writer was unable to attend. The Grand Banquet was given at the Jefferson Hotel, at which some three hundred, including the writer, took part. A pleasant feature of this, and one worthy of future imitation, was the presence of ladies, the guests of the Congress. It was two A.M. before the long list of speakers, representing all civilized countries, had concluded the post-prandial work.

It is with profound regret that it is impossible to review the work of this Congress with any feelings of satisfaction. It failed, in the opinion of the writer, to represent the dentistry of America as he understands it, and it failed to give expression to that broader international dentistry of which we hear so much and know, intimately, so little. The undignified course pursued at the opening seemed to tinge the whole Congress, and if the residents of foreign countries return to their several homes with unfavorable opinions, it will be due to some who were unable to rise to the standard that the world demands of a professional cultured gentleman.

This article cannot be closed without entering a decided protest against the method adopted of having a door-tender to keep out all persons who failed to wear a button, the sign of membership. This senseless order resulted in several wordy conflicts with members, anything but creditable. Dental congresses are, in the estimation of the writer, educational bodies, or should be so, and the young men in the profession, or undergraduates, should be freely invited to take a listener's part. Not many of these can afford the ten dollars necessary to become a member. It seemed as though the committee, in ordering this, carried this Congress back to the middle of the last century when "No Admittance" was figuratively over every office door. This was not the least of the mistakes made in this Congress, and it certainly was one of the most unpleasant. The science worthy to be called by that name should be free to all able to understand.

It is with profound regret and with a sense of humiliation that nothing better can be said of the visible result of this Congress. We in this country had looked forward to it as a means of extending the professional view throughout the world, thus making our lives broader through a closer union with the dentists of modern civilization. In some instances this was secured, but upon the whole the true cosmopolitan spirit was not advanced, neither were we, to any great extent, aided by foreigners in the discussions. In the papers this was different, many of the most valuable being from the hands of foreign writers. It was, however, essentially an English-speaking congress, the international feature being almost entirely lost.

It is not probable that another International Dental Congress will be held in the very near future, but if such should be in contemplation, it is to be hoped that those who manage it will have learned to avoid the unfortunate errors committed by the various committees that managed the International Dental Congress at St. Louis, 1904.

